# Potential TMA-Producing Bacteria Are Ubiquitously Found in Mammalia

**DOI:** 10.3389/fmicb.2019.02966

**Published:** 2020-01-09

**Authors:** Silke Rath, Tatjana Rud, Dietmar H. Pieper, Marius Vital

**Affiliations:** ^1^Microbial Interactions and Processes Research Group, Helmholtz Centre for Infection Research, Braunschweig, Germany; ^2^Institute for Medical Microbiology and Hospital Epidemiology, Hannover Medical School, Hanover, Germany

**Keywords:** trimethylamine, choline, carnitine, betaine, gut microbiota, diet, mammals, microbial ecology

## Abstract

Human gut bacteria metabolize dietary components such as choline and carnitine to trimethylamine (TMA) that is subsequently oxidized to trimethylamine-*N*-oxide (TMAO) by hepatic enzymes. Increased plasma levels of TMAO are associated with the development of cardiovascular and renal disease. In this study, we applied gene-targeted assays in order to quantify (qPCR) and characterize (MiSeq) bacterial genes encoding enzymes responsible for TMA production, namely choline-TMA lyase (*CutC*), carnitine oxygenase (*CntA*) and betaine reductase (*GrdH*) in 89 fecal samples derived from various mammals spanning three dietary groups (carnivores, omnivores and herbivores) and four host orders (Carnivora, Primates, Artiodactyla and Perissodactyla). All samples contained potential TMA-producing bacteria, however, at low abundances (<1.2% of total community). The *cutC* gene was more abundant in omnivores and carnivores compared with herbivores. C*ntA* was almost absent from herbivores and *grdH* showed lowest average abundance of all three genes. Bacteria harboring *cutC* and *grdH* displayed high diversities where sequence types affiliated with various taxa within *Firmicutes* dominated, whereas *cntA* comprised sequences primarily linked to *Escherichia*. Composition of TMA-forming communities was strongly influenced by diet and host taxonomy and despite their high correlation, both factors contributed uniquely to community structure. Furthermore, Random Forest (RF) models could differentiate between groups at high accuracies. This study gives a comprehensive overview of potential TMA-producing bacteria in the mammalian gut demonstrating that both diet and host taxonomy govern their abundance and composition. It highlights the role of functional redundancy sustaining potential TMA formation in distinct gut environments.

## Introduction

The metabolite trimethylamine (TMA) is produced by various gut microbial taxa from dietary quaternary ammonium compounds, mainly choline (Wang et al., 2011) and carnitine (Koeth et al., 2013). Subsequently, it is absorbed by the intestinal epithelium and oxidized by host hepatic flavin monooxygenases to trimethylamine *N*-oxide (TMAO), which is associated with the development of cardiovascular and kidney disease (Tang et al., 2013, 2015). Several studies have investigated the impact of host factors, in particular diet and individual dietary compounds, on human TMAO plasma levels (summarized in Supplementary Table S1), where increased TMAO levels were observed in subjects on an animal-product based diet (Tang et al., 2013; Krüger et al., 2017; Wang et al., 2018) compared with vegetarians (Koeth et al., 2013, 2019). In general, TMA concentrations are dependent on the amount of available precursors and the abundance and activity of bacteria catalyzing TMA formation (Rath et al., 2018), which are in competition with the host that absorbs those precursors as essential nutrients. Choline is converted to acetylcholine, a crucial neurotransmitter (Zeisel and Blusztajn, 1994), whereas carnitine plays an important role in the fatty acid β-oxidation (Rebouche and Seim, 1998). The individual contribution of these factors to TMA(O) levels remain elusive, partly because quantification of TMA producers in the gut are largely lacking. It is, hence, crucial to understand the distribution of those bacteria in the gut and specify their niches in order to design effective and sustainable treatment strategies minimizing TMAO plasma concentrations (Rath et al., 2018).

We have previously developed assays targeting key genes encoding enzymes responsible for TMA formation from choline (choline-TMA lyase, *CutC*) and carnitine (carnitine monooxygenase, *CntA*), in order to explore their abundance and diversity in human samples, where we observed a diverse community harboring those genes, however, at low concentrations, encompassing less than 1% of the total bacterial community (Rath et al., 2017). In this study, we explored the distribution and abundance of *cutC* and *cntA* in fecal matter of various mammals, spanning four taxonomic orders (Carnivora, Primates, Artiodactyla, and Perissodactyla, with the latter two belonging to the Ungulata clade) and covering three different diets (carnivorous, omnivorous, and herbivorous). Moreover, we expanded our assays to target the gene encoding the subunit B of betaine reductase (*grdH*) that catalyzes TMA formation from betaine (Andreesen, 1994). The animals greatly differed in their dietary habits, gut anatomy and physiology, providing a broad range of distinct gut environments that enable insights into major factors governing the distribution of TMA-producing bacteria in that habitat. Diet and host phylogeny are known to control gut microbiota structure (De Filippo et al., 2010; Wu et al., 2011; David et al., 2014; Hicks et al., 2018) and functionality in mammals (Groussin et al., 2017; Youngblut et al., 2018), however, knowledge on how they influence abundance and composition of TMA-forming bacteria specifically is not available.

## Materials and Methods

### Sample Collection, DNA Extraction and Gene-Targeted Assays

Fecal samples from mammals were collected at the “Arche Noah” Zoo (Braunschweig, Germany), Serengeti Park (Hodenhagen, Germany) and Wisentgehege Springe (Springe, Germany) within 24 h after defecation into tubes containing RNAlater (roughly twice the sample volume; Sigma-Aldrich, Munich, Germany) and stored at −80°C. The HZI Animal Welfare Committee did not require the study to be reviewed or approved by an ethics committee because it does not involve any procedures on animals.

Metadata is presented in Supplementary Table S2. Dietary classification was based on the dietary regime of individual sites. Species were mostly represented by one or two animals, whereas for lions seven samples from the same species were included (Supplementary Table S2). DNA extraction, gene quantification by quantitative PCR (qPCR), amplicon library preparation and sequencing of the 16S rRNA gene, *cutC* and *cntA* amplicons were performed with degenerate primers (Supplementary Table S3) as previously described (Rath et al., 2017) with small modifications: DNA template concentration was decreased to 10 ng and annealing temperature was reduced to 55°C during preparation of 16S rRNA gene libraries. Sequencing was performed on Illumina MiSeq platform (2 × 300 bp). Degenerate primers (final concentration of 1.5 μM each) targeting *grdH* encoding the β-subunit of the betaine reductase were designed covering the majority (79%, allowing for 1 mismatch in each primer) of unique sequences of our previously constructed database (Rath et al., 2018) and are shown in Supplementary Table S3. Conditions for *grdH*-specific qPCR and amplicon generation were the same as for *cutC* and *cntA* and an annealing temperature of 53°C was applied. The purification step after the first PCR was omitted for *grdH* due to negligible primer dimer formation. Primers were synthesized and HPLC purified by Eurofins Genomics (Ebersberg, Germany). Standard curves for qPCR were generated from dilutions of 10^6^ to 10^3^ genome copies using the genomic sequence of *Clostridium bolteae* (DSM 15670). A quantification limit of 10^3^ gene copies per reaction was determined. Previously published data on *cutC* and *cntA* of human samples (Rath et al., 2017) were included and the same samples were analyzed for *grdH* in this study (*N* = 48; for samples #19 and #40 no material was available). The qPCR results of functional genes were normalized to the total 16S rRNA gene abundance (Rath et al., 2017).

### Bioinformatic Procedures

Bioinformatic analyses of functional gene data were performed as described previously (Rath et al., 2017), involving several quality-filtering steps and clustering (complete linkage) of translated sequences at 90% amino acid similarity using tools from the ribosomal Database Project (RDP) (Cole et al., 2014). All sequences that passed FrameBot analysis and displayed *bona fide* reference sequences as closest match were included into downstream analyses. Before clustering, sequences were sampled to an equal depth per sample [11,000 (*cutC*), 10,000 (*cntA*) and 25,000 (*grdH*)]. 16S rRNA gene sequences were treated as described before (Rath et al., 2017). Raw sequences were submitted to the European Nucleotide Archive (ENA) under accession number PRJEB34622.

### Statistical Analyses

Illustrations of gene abundances and statistics (Kruskal–Wallis test with subsequent Benjamini-Hochberg false discovery rate (FDR) correction for multiple comparison) on abundance differences were performed in GraphPad Prism (version 8.2.0; Graph-Pad Software, San Diego, CA, United States). All other statistical analyses were performed in R (v. 3.5.2). The Kruskal–Wallis test (*kruskal.test*) with subsequent FDR correction (*p.adjust* (method = “fdr”) (based on Benjamini-Hochberg procedure)) was applied to assess differences in taxa and gene clusters between groups. Permutational analysis of variance (permANOVA) (function *vegan:adonis*) was performed to deduce significant differences between groups of samples with or without adjustment for either parameter, diet and host phylogeny. Groups of samples were considered significantly different if the relative *p*-value was <0.05. Stacked bar and bubble plots depicting gene abundances affiliated with individual taxa were constructed in R with ggplot2 (v. 3.2.0). Catabolic genes data was binned at genus level based on RDP taxonomy of closest matching reference as described previously in Rath et al. (2017). References that derive from genomes not assigned a genus were binned at the family level. Since many *cutC* sequences were most similar to a reference sequence previously described in an unclassified *Clostridiales* (PATRIC genome ID: 165185.5) those results are shown separately. Similarly, for *grdH*, sequences linked to two unclassified *Lachnospiraceae* (based on RDP), termed *Dorea* sp. AGR 2135 (PATRIC genome ID: 1280669.3) and *Coprococcus* sp. HPP 0048 (PATRIC genome ID: 1078091.3) within NCBI, were treated separately from sequences previously observed in other unclassified *Lachnospiraceae* references. Data matrices comprising square-root transformed count data of catabolic gene clusters or 16S rRNA gene abundances (on genus level) were used to construct sample-similarity matrices using the Bray-Curtis algorithm in PRIMER (Version 7.0.7) (Clarke et al., 2014). Non-metric multidimensional scaling (nMDS) plots were constructed in PRIMER. The function MVDISP in PRIMER was used to calculate the multivariate dispersion within distinctive groups.

Random Forest (RF) analysis was performed in R with the function *caret:train* (method = “ranger”) applying a five times repeated fivefold cross-validation strategy. Feature selection was based on abundance (≥0.1% mean abundance and presence in ≥25% of samples), statistic [*p* < 0.001 differentially distributed between all groups including humans (kruskal.test)], correlation (features displaying a value >0.5 *caret:findCorrelation* were omitted) and *boruta* analysis in R. Finally, 23 and 20 taxa based on the 16S rRNA gene, 24 and 27 *cutC* clusters and 13 and 13 *grdH* clusters were used for generation of models for diet and host phylogeny, respectively. Results from humans were included into the analyses as a separate group.

## Results and Discussion

### Gut Microbiome Profiles of Mammals Differed Between Dietary and Phylogenetic Groups

**FIGURE 1:**
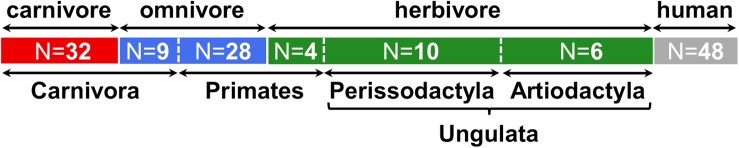
Overview of samples included in this study. Numbers (N) of animals belonging to each group are indicated. At the top, the dietary classification is shown and highlighted by colors (red: carnivore, blue: omnivore, green: herbivore, gray: human), while at the bottom the taxonomic affiliations at the order (and clade) level is given. More detailed information is presented in Supplementary Table S2.

In the current study, we investigated gut microbiota with specific interest on the TMA-forming potential of fecal samples (*N* = 89) from various mammals, comprising 51 different species from the four orders Carnivora, Primates, Artiodactyla and Perissodactyla (Figure 1 and Supplementary Table S2). In the following analyses, animals belonging to Artiodactyla and Perissodactyla were summarized as Ungulata. Of the 41 Carnivora, nine were characterized by an omnivorous lifestyle whereas four of the typically omnivorous Primates were fed a herbivorous diet. Data from humans (*N* = 48) were included for comparison.

**FIGURE 2:**
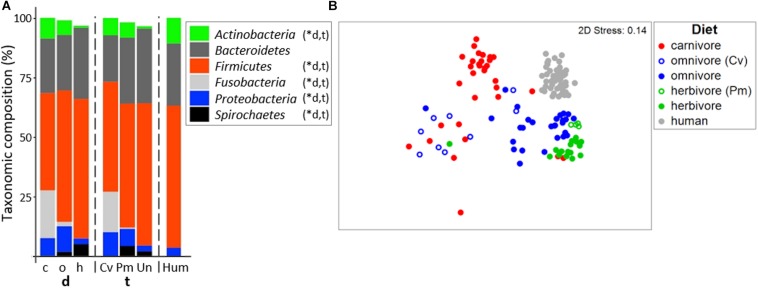
Bacterial community composition in mammals. **(A)** shows relative mean abundances of bacterial phyla (based on 16S rRNA gene abundances) for the dietary (d) groups carnivores (c), omnivores (o), and herbivores (h) as well as the taxonomic (t) groups Carnivora (Cv), Primates (Pm), and Ungulata (Un). The community composition of human (Hum) samples is shown for comparison. ^∗^Denotes significant difference (*p* < 0.05) in relative abundance between dietary and taxonomic groups as calculated by FDR-corrected Kruskal–Wallis tests. Only phyla with mean abundances >1% are shown. The non-metric multi-dimensional scaling plot (nMDS) in **(B)** depicts the ordination results of gut communities based on Bray-Curtis similarities of standardized square-root transformed relative abundance data at the genus level.

In accordance with previous studies (Ley et al., 2007; Costa et al., 2012), the gut microbiome of all mammalian species analyzed was dominated by *Firmicutes* and *Bacteroidetes* (Figure 2A; for details on all individual samples see Supplementary Figure S1). On average, a high relative abundance of *Fusobacteria* was only observed in carnivores (20.0 ± 16.8%) and Carnivora (16.9 ± 16.4%), as previously described (Nelson et al., 2013; Zhu et al., 2018). Similar to other reports (Ley et al., 2008; Vital et al., 2015), community structure clustered according to diet (PermANOVA test, *R*^2^ = 0.273, *p* ≤ 0.001) and host taxonomy (*R*^2^ = 0.310, *p* ≤ 0.001) where both factors were tightly coupled (Figure 2B). PermANOVA analyses adjusting for each factor remained significant (*R*^2^ = 0.074, *p* ≤ 0.001 for diet and *R*^2^ = 0.111, *p* ≤ 0.001 for taxonomy) indicating that both factors independently shape community composition explaining in total 38.3% of the variance. In particular, gut microbiota of carnivores grouped separately from those of herbivores in nMDS analyses. Gut microbiota composition of carnivores and omnivores showed higher within-group variation (multivariate dispersion (MVDISP) factors of 1.251 and 1.439, respectively) compared with herbivores and humans that exhibited MVDISP factors of 0.846 and 0.663, respectively (Figure 2B).

Based on their total microbiome structure, omnivorous Carnivora grouped according to taxonomy and not with their individual dietary partners (Figure 2B). Accordingly, the bacterial community structure of omnivorous Carnivora was more similar to that of carnivorous Carnivora (mean Bray-Curtis similarity (BC) of 31.6 ± 10.9%) than to that of omnivorous Primates (mean BC of 22.6 ± 10.9%, *p* < 0.001 according to the FDR-corrected Kruskal–Wallis test). In contrast, gut microbiota composition of herbivorous Primates is less similar to those of other Primates (mean BC of 44.9 ± 13.8%) compared with Ungulata’s microbiota (mean BC of 54.6 ± 10.8%, *p* < 0.01 using Kruskal–Wallis test). Our observations suggest that taxonomy is driving gut microbiota composition in Carnivora as previously reported (Ley et al., 2008; Vital et al., 2015), whereas diet is a major factor within Primates.

Random Forest models were built in order to distinguish between dietary or host taxonomic groups. Classification was possible, yielding classification accuracies of 0.91 and 0.96 for diet and host taxonomy, respectively. Specifically, four omnivorous Carnivora (*N* total of 9) were wrongly detected as carnivorous (racoon dog, fox, skunk, and small-clawed otter) by the diet model, whereas all four herbivorous Primates were correctly classified by both models. The importance of bacterial genera for classification differed between the two models demonstrating that genera distinctly contributed to the differentiation between groups. For instance, *Bacteroides* and *Dialister* were the top discriminators for diet, whereas those genera played a minor role for differentiation between host taxonomies, where *Clostridium* XI was much more important (Supplementary Table S4). Those results further indicate that both factors, while highly correlated, uniquely contribute to the composition of gut microbiota.

### Genes Encoding TMA-Forming Enzymes Are Ubiquitously Present in Mammals but at Low Abundances

**FIGURE 3:**
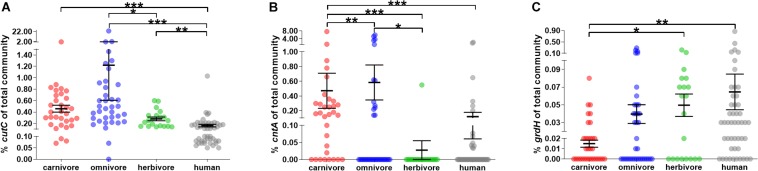
Relative abundance of genes encoding enzymes for TMA formation in mammals. **(A–C)** show the abundance of *cutC*, *cntA* and *grdH* of the total bacterial community (relative to total 16S rRNA gene abundance), respectively. In all panels, samples were grouped into herbivores (green), omnivores (blue) and carnivores (red). Human samples (gray) are shown for comparison. The black line represents the mean and its standard error. Significant differences based on FDR-corrected Kruskal–Wallis tests are symbolized as follows: ^∗^*p* ≤ 0.05; ^∗∗^*p* ≤ 0.01; ^∗∗∗^*p* ≤ 0.001. Note the *y*-axis breaks at 0.1 and 1.5% for *cutC* abundance, at 0.1 and 1% for *cntA* abundance and 0.02 and 0.1% for *grdH* abundance.

In all fecal samples included in this study at least one of the three genes encoding the key enzymes of pathways catalyzing TMA formation, namely, choline TMA lyase (*cutC*), carnitine oxygenase (*cntA*) and betaine reductase (*grdH*), was detected (Figure 3; for detailed results see Supplementary Figure S2).

*CutC* was ubiquitously present in all animal samples, except for one ferret. In contrast, *cntA* and *grdH* were detected in 42.7 and 55.1% samples, respectively (Figure 3 and Supplementary Figure S2). Those results are in line with data derived from human subjects, where *cutC* was previously found in all samples, whereas *cntA* was detected in only 26.0% (Rath et al., 2017), while *grdH* was found in 79.2% of human samples analyzed. Previous metagenome-based analyses of human data detected functional genes in a similar amount of samples with *cutC* detected in 95.7 and 99.7%, *cntA* in 33.5 and 14.8%, and *grdH* in 80.6 and 94.1% samples (dataset XII and XIII, respectively) (Rath et al., 2018).

The overall relative abundance of potential TMA producers was low in most animals (average cumulative abundance of 1.19 ± 0.29% (SEM) of total bacterial community) and averages of *cutC* (0.73 ± 0.26%) and *cntA* (0.42 ± 0.13%) showed higher abundances compared with *grdH* (0.03 ± 0.01%) (Kruskal–Wallis test with FDR correction, *p* ≤ 0.001 and *p* ≤ 0.05, respectively) (Figure 3). Observed lower abundances of *grdH* is in accordance with metagenomic data derived from human samples (Jameson et al., 2016; Rath et al., 2018). In comparison to the other genes, *cntA* was highly abundant in a few samples (five samples exhibited ≥3% of bacteria harboring that gene), which is in accordance with the pattern previously observed in humans, where specific samples displayed communities that contained ≥1% of bacteria carrying *cntA* (Rath et al., 2017). One common marmoset had the highest TMA-formation potential, with 23.1 and 5.2% of bacteria of its total fecal microbiota harboring *cutC* and *cntA*, respectively. However, this high abundance is not common to this species as the second animal exhibited a *cutC* gene abundance of 0.62% and *cntA* was below the quantification limit.

Dietary groups differed significantly in their *cutC* and *cntA* abundances (Kruskal–Wallis test with FDR correction, *p* < 0.001 and *p* < 0.001, respectively). More specifically, *cutC* was more abundant in omnivores (1.22 ± 0.61%) than in herbivores (0.28 ± 0.03%), whereas *cntA* was of highest abundance in carnivores (0.57 ± 0.25%) and absent from herbivores (except the sample derived from the camel) suggesting the precursor carnitine as a selection factor for *cntA*-harboring communities since herbivores are fed only minute amounts of carnitine. In contrast, *grdH* was lower abundant in carnivores (0.01 ± 0.00%) compared with herbivores (0.05 ± 0.01%).

### *Firmicutes* Dominate the Highly Diverse *CutC* Community in Mammals

**FIGURE 4:**
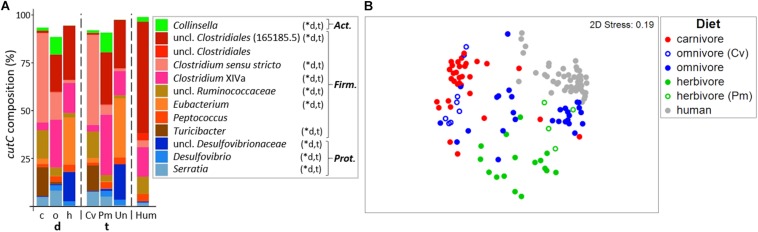
Taxonomic composition of *cutC* genes in mammals. **(A)** shows the relative mean abundance of *cutC* gene types (binned according to affiliated taxa) for the dietary (d) groups carnivores (c), omnivores (o), and herbivores (h), as well as the taxonomic (t) groups Carnivora (Cv), Primates (Pm), and Ungulata (Un). *CutC* gene variants observed in human (Hum) samples are shown for comparison. ^∗^Denotes significant difference in relative abundance between dietary and taxonomic groups as calculated by FDR-corrected Kruskal–Wallis tests. *Act*., *Actinobacteria*; *Firm*., *Firmicutes*; *Prot*., *Proteobacteria.* Only taxa with mean abundances >1% are shown. The non-metric multi-dimensional scaling plot (nMDS) in **(B)** depicts the ordination results of *cutC* gene communities based on Bray-Curtis similarities of standardized square-root transformed abundance data from complete linkage clustering (90% similarity of translated protein sequences).

In total, 429 different *cutC* sequence clusters (based on 90% amino acid similarity) were detected with an average of 48 ± 12 clusters per sample. Sequences were associated with three distinct phyla, namely *Firmicutes*, *Actinobacteria*, and *Proteobacteria* (Figure 4A; for details on all individual samples see Supplementary Figure S3). Sequence types affiliated with multiple taxa within *Firmicutes* dominated in all groups, whereas *cutC* genes similar to those previously observed in *Collinsella* (*Actinobacteria*) and *Serratia* as well as *Desulfovibrionaceae* (both *Proteobacteria)* were also found. *CutC* sequences had high similarity to references, where 94.2%/99.9% comprised clusters, whose representative sequence displayed >90%/>70% similarity to a reference sequence.

Similar to the results derived from 16S rRNA gene analysis, the *cutC* gene community structures differed significantly between both dietary (PermANOVA test, *R*^2^ = 0.201, *p* ≤ 0.001) and taxonomic (*R*^2^ = 0.225, *p* ≤ 0.001) groups. Both factors combined were able to explain 27.2% of community variations (*R*^2^ = 0.272). Despite their high correlation, adjusting for each factor still yielded significant results (*R*^2^ = 0.047, *p* ≤ 0.001 and *R*^2^ = 0.071, *p* ≤ 0.001 for diet and taxonomy, respectively), indicating that both factors independently contribute to the *cutC* gene sequence composition.

The *cutC* gene pools of herbivorous and carnivorous animals were clearly separated on the nMDS plot and samples displayed lower dispersion (MVDISP factors of 1.261 and 1.071, respectively) than samples from omnivores (MVDISP factor of 1.398) (Figure 4B). Human *cutC* communities formed a separate group displaying lowest dispersion (MVDISP factor of 0.690) and were most similar to certain Primates, namely Angola colobi, baboons, barbary macaques, chimpanzees, lion-tailed macaques, and siamangs, respectively, underlining the influence of host taxonomy on *cutC* community composition.

The *cutC* gene pool in humans was clearly dominated by sequences similar to that previously observed in an unclassified *Clostridiales* (PATRIC genome ID 165185.5). Those sequences were less abundant in Primates and Ungulata and almost absent in Carnivora (Figure 4A). The latter were dominated by sequences linked to *Clostridium sensu stricto*, unclassified *Ruminococcaceae* and *Turicibacter*. Sequences similar to those previously observed in *Clostridium* XIVa were detected at high abundances in omnivores, herbivores and humans, while herbivores were additionally enriched in sequence types associated with *Eubacterium* spp. and unclassified *Desulfovibrionaceae*. *CutC* communities of the two carnivorous American black bears were largely composed of sequences previously observed in members of *Clostridium* XIVa (∼50%), which were almost absent in other Carnivora. This is in accordance with the results of their total bacterial communities being more similar to those of omnivorous and herbivorous animals than to those of other carnivores (Figure 2 and Supplementary Figure S1). In contrast, previous reports described the black bear as an omnivorous animal (Ley et al., 2008; Muegge et al., 2011; Song et al., 2017) that was characterized by Carnivora-like microbial communities (Ley et al., 2008; Muegge et al., 2011).

Omnivorous Carnivora exhibited *cutC* gene variants more similar to those of carnivorous Carnivora (mean BC of 20.2 ± 13.8%) than to those of omnivorous Primates (mean BC of 7.3 ± 10.0%, *p* < 0.001 using FDR-corrected Kruskal–Wallis test), indicating host taxonomy as a major factor selecting *cutC* sequence types, which is supported by a close grouping of *cutC* communities of omnivorous Primates with herbivorous Primates in the nMDS plot (Figure 4B). However, the similarity between *cutC* communities of herbivorous and omnivorous Primates (mean BC of 19.3 ± 15.1%) was not significantly different from the ones between herbivorous Primates and Ungulata (mean BC of 15.6 ± 10.3%; *p* > 0.05 using FDR-corrected Kruskal–Wallis test).

Overall, animals of the same species were characterized by similar TMA-producing communities (mean BC of 55.2 ± 14.8%), even if sampled at different locations (Supplementary Figure S3), indicating that individual host species select for specific *cutC* gene-types.

We established RF models based on *cutC* sequence types in order to classify the samples analyzed into their dietary and taxonomic groups. Similar to the results of total microbial communities, RF models could differentiate between groups for both diet and host taxonomy as both displayed high classification accuracies of 0.88. Sequence variants with the highest importance were similar in both models, except for sequence type c_079, associated with *Clostridium sensu stricto*, having a minor importance for the classifier based on the diet model (Supplementary Table S4). Five of the omnivorous Carnivora were classified into their respective dietary group, whereas the remaining three were incorrectly classified as carnivorous Carnivora (raccoon dog, raccoon and fox). Two of the four herbivorous Primates were correctly classified only by diet (gray langurs), while the other two were only correctly classified according to their taxonomy (Angola colobi).

### *CntA* Genes Originating From *Escherichia* Dominate the *CntA*-Containing Community in All Mammals

**FIGURE 5:**
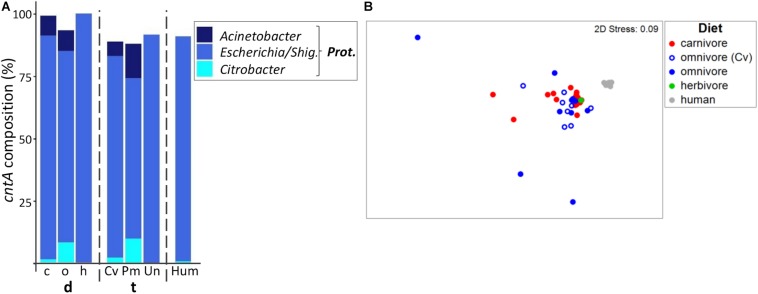
Taxonomic composition of *cntA* genes in mammals. **(A)** shows the relative mean abundance of *cntA* gene types (binned according to affiliated taxa) for the dietary (d) groups carnivores (c), omnivores (o), and herbivores (h) as well as the taxonomic (t) groups Carnivora (Cv), Primates (Pm), and Ungulata (Un). *CntA* gene variants observed in human (Hum) samples are shown for comparison. *Prot*., *Proteobacteria*; *Shig*., *Shigella*. Only taxa with mean abundances >1% are shown. The non-metric multi-dimensional scaling plot (nMDS) in **(B)** depicts the ordination results of *cntA* gene communities based on Bray-Curtis similarities of standardized square-root transformed abundance data from complete linkage clustering (90% similarity of translated protein sequences).

Diversity of *cntA* gene-types was lower compared with *cutC* and *grdH* (Supplementary Figure S4), and on average 22 ± 9 distinct clusters that were all similar to those previously observed in *Proteobacteria*, in particular *Escherichia* (Figure 5A; for details on all individual samples see Supplementary Figure S5) were detected. Sequences had high similarity to references, namely, 96.4%/98.7% of them comprised clusters, whose representative sequence displayed >90%/>70% similarity to a reference sequence.

Based on permANOVA analysis, diet and taxonomy in combination were able to explain only 10% of community variations (*R*^2^ = 0.098, *p* ≤ 0.05). After adjustment for each factor, differences between dietary and taxonomic groups became non-significant (both *p* > 0.05) with *R*^2^ = 0.047 and *R*^2^ = 0.025 for diet and taxonomy, respectively.

Humans mostly contained *cntA* genes originating from *Escherichia* (Rath et al., 2017), whereas both carnivores and omnivores additionally harbored sequences linked to *Acinetobacter* spp. and *Citrobacter* spp. (Figure 5A). In herbivores, *cntA* was detected only in one sample (camel), which was dominated by sequences affiliated with *Escherichia*. The nMDS plot showed no clear separation between *cntA* gene pools of carnivores and omnivores and both groups had high dispersions (MVDISP factors of 0.936 and 1.458), whereas humans formed a separate cluster displaying lower dispersion (MVDIP factor of 0.232) (Figure 5B).

*CntA* communities of omnivorous Carnivora were more similar to those of carnivorous Carnivora (mean BC of 68.1 ± 12.0%) than to those of omnivorous Primates (mean BC of 54.7 ± 20.8%, *p* ≤ 0.001 based on FDR-corrected Kruskal–Wallis test), indicating that also for *cntA* host taxonomy contributed to community composition to a higher extend than diet.

The RF model based on taxonomy revealed higher accuracy (accuracy of 0.85) than the diet model (accuracy of 0.69), which is explained by omnivorous Carnivora, where five (*N* total = 9) were misclassified as carnivores. It should be noted that the use of RF analysis for *cntA* remains questionable since only a few samples per group could be included into calculations.

### *Firmicutes* Also Dominate the Diverse *GrdH* Community

**FIGURE 6:**
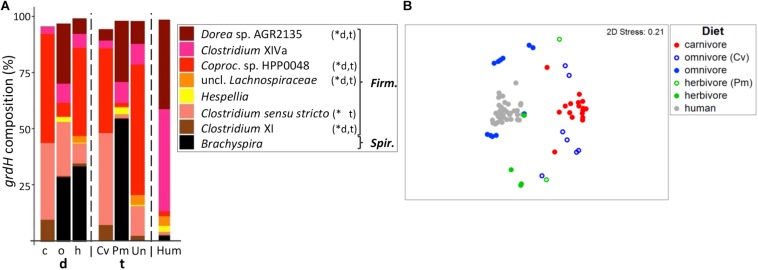
Taxonomic composition of *grdH* genes in mammals. **(A)** shows the relative mean abundance of *grdH* gene types (binned according to affiliated taxa) for the dietary (d) groups carnivores (c), omnivores (o), and herbivores (h) as well as the taxonomic (t) groups Carnivora (Cv), Primates (Pm), and Ungulata (Un). *GrdH* gene variants observed in human (Hum) samples are shown for comparison. ^∗^Denotes significant difference in relative abundance between dietary and taxonomic groups as calculated by FDR-corrected Kruskal–Wallis tests. *Firm, Firmicutes; Spir*., *Spirochetes*; *Coproc*., *Coprococcus.* Only taxa with mean abundances >1% are shown. The non-metric multi-dimensional scaling plot (nMDS) in **(B)** depicts the catabolic *grdH* gene structures based on Bray-Curtis similarities of standardized square-root transformed abundance data from complete-linkage clustering (90% similarity of translated protein sequences).

Similar to *cutC*, we observed a highly diverse *grdH* sequence pool with 52 ± 28 clusters per sample comprising a total of 577 clusters. *GrdH* sequences were dominated by variants similar to those previously identified in various members of *Firmicutes*, whereas some linked to *Spirochetes*, specifically *Brachyspira*, were detected as well (Figure 6A; for more details see Supplementary Figure S6). Sequences had high similarities to references, where 74.3%/98.6% comprised clusters, whose representative sequence displayed >90%/>70% similarity to a reference sequence.

PermANOVA analyses revealed both diet (*R*^2^ = 0.251, *p* ≤ 0.001) and host taxonomy (*R*^2^ = 0.239, *p* ≤ 0.001) as significant discriminators, even if adjusted for each other (*R*^2^ = 0.151, *p* ≤ 0.001 and *R*^2^ = 0.163, *p* ≤ 0.001 for diet and taxonomy, respectively). Together the two factors were able to explain 40.2% of community variations.

*GrdH* communities of carnivores were characterized by a MVDISP of 1.087 forming a group in nMDS analysis. In comparison, omnivores and herbivores scattered throughout the nMDS plot and displayed higher dispersion values (MVDISP factors of 1.641 and 1.376, respectively) (Figure 6B). Datapoints of non-carnivorous Carnivora clearly grouped with other taxonomic members, where *grdH* communities of omnivorous Carnivora were more similar to those of carnivorous Carnivora (mean BC of 8.8 ± 8.2%) than to those of omnivorous Primates (mean BC of 1.7 ± 2.1%, *p* < 0.001 according to Kruskal–Wallis test). In comparison, *grdH* communities of herbivorous Primates were highly dissimilar to both those of other herbivores (mean BC of 3.1 ± 2.3%) and those of omnivorous Primates (mean BC of 1.0 ± 0.8%, *p* > 0.05 using FDR-corrected Kruskal–Wallis test). Similar to the results of the other genes encoding TMA-forming enzymes, *grdH* sequences derived from humans formed their own cluster in the nMDS plot.

*GrdH* communities of carnivores were mainly comprised of genes similar to those previously observed in *Clostridium sensu stricto* and *Coprococcus* sp. HPP0048 and either taxon dominated individual samples (Supplementary Figure S6). Omnivores were characterized by *grdH* sequences associated with *Dorea* sp. AGR2135, *Clostridium* XIVa and *Brachyspira.* The latter was highly abundant in herbivorous and omnivorous Primates, except for the tamarins. In humans, we observed two *grdH* community types that were either dominated by sequences linked to *Dorea* sp. AGR2135 (*N* = 14) or by members of *Clostridium* XIVa (*N* = 23) (Supplementary Figure S6). Results are in accordance with a previous report (Borrel et al., 2017) as well as our own screening of metagenomic data (Rath et al., 2018), which both revealed members of *Clostridium* XIVa and *Dorea* as the most abundant *grdH*-containing bacteria of human microbiota.

Random Forest analyses could discern between dietary and taxonomic groups yielding high accuracies (both 0.91). The importance of sequence variants to discriminate between groups were different in each model. For example, the sequence type c_367 (linked to *Clostridium sensu stricto*) was more important for diet, while sequence type c_211 (associated with *Coprococcus* sp. HPP 0048) contributed highly to the taxonomy model (Supplementary Table S4). Two omnivorous Carnivora (*N* total of seven) and the two herbivorous Primates (*N* total of two) were wrongly classified by the diet model.

## Conclusion

We detected potential TMA-producing communities in fecal samples from all animals tested, demonstrating that this functional group is omnipresent in the gut across Mammalia. However, those bacteria occupy only a small niche as their abundance was low in all samples (mean cumulative relative abundance of ≤1.2% of total community), except for *cntA* that was detected at high concentrations in a few samples. In humans, a similar abundance pattern was previously observed (Rath et al., 2017, 2018). Diversity of TMA-forming gene-types was high, especially for *cutC* and *grdH*, and associated with many taxa of several distinct phyla. Composition clearly differed between groups, and both diet and phylogeny governed the abundance and composition of TMA-producing bacterial communities. Despite their high correlation, each factor independently contributed to community structure. Overall, our data highlights the role of functional redundancy of taxonomically distinct, potential TMA producers leading to their presence in all gut environments tested here. Results demonstrate that potentially TMA-forming communities adapt to various nutritional conditions suggesting that nutrition-based interventions in humans might not be able to completely eradicate those bacteria from the gut. Nevertheless, differences in abundance between groups were detected. For instance, both *cutC* and *cntA* were less abundant in herbivores compared with omnivores or carnivores indicating that a plant-based diet might indeed reduce the abundance of bacteria exhibiting those genes giving a possible explanation for reduced TMAO plasma levels observed in human subjects that foster a vegetarian life-style (Supplementary Table S1). Since precursor concentrations are also reduced in herbivorous diets, conclusions on the impact of the abundance of TMA forming bacteria on TMA(O) concentrations are speculative. Still, the absence of *cntA* from herbivores is intriguing and suggests a tight correlation between the precursor carnitine, which is primarily found in red meat (Steiber et al., 2004), and abundance of bacteria harboring this gene. Nevertheless, we have previously observed that *cntA* is not expressed in human fecal samples (Rath et al., 2018), which is in accordance with results of another study that did not find correlations between *cntA* gene abundance and TMAO plasma levels (Wu et al., 2018), and the role of *cntA*-containing bacteria for TMA formation in the colon, thus, needs further investigations. In fact, Koeth et al. (2019) have recently reported an additional mechanism of gut microbiota converting carnitine to TMA via a two-step process involving the intermediate γ-butyrobetaine (γBB) and demonstrated this pathway as the major route for TMA formation from carnitine in the human gut. Interestingly, the second step catalyzing the reaction from γBB to TMA was reported to be diet-inducible with a markedly lower extent in vegans/vegetarians compared with omnivores. Since genes involved in the latter step are unknown we did not include this pathway in our study. It should be noted that we consider the elucidation of microbial TMA-formation pathways as an ongoing process that needs continuing effort in order to get a holistic picture on bacteria catalyzing this important reaction and to design effective intervention strategies.

In summary, this study gives a comprehensive overview of potential TMA-producing bacteria in the mammalian gut demonstrating that both diet and host taxonomy govern their abundance and composition.

## Data Availability Statement

The datasets generated for this study can be found in the PRJEB34622.

## Ethics Statement

The HZI Animal Welfare Committee did not require the study to be reviewed or approved by an ethics committee because it does not involve any procedures on animals.

## Author Contributions

MV and SR contributed to the concept. SR and TR contributed to the laboratory work. MV contributed to the bioinformatic analysis. SR, MV, and DP contributed to the data analysis and wrote the manuscript. All authors read and approved the final version of the manuscript.

## Conflict of Interest

The authors declare that the research was conducted in the absence of any commercial or financial relationships that could be construed as a potential conflict of interest.
