# Supplementary material for: Potential TMA-Producing Bacteria Are Ubiquitously Found in Mammalia
Source: Front Microbiol. 2020 Jan 9;10:2966. doi: 10.3389/fmicb.2019.02966 (PMC6964529; doi:10.3389/fmicb.2019.02966)
Supplement: Supplementary file 1 [file Data_Sheet_1.PDF]

Supplementary Material

Potential TMA-producing Bacteria are Ubiquitously Found in Mammalia

Silke Rath, Tatjana Rud, Dietmar H. Pieper, Marius Vital*

***Correspondence:**

Marius Vital

Vital.Marius@mh-hannover.de

1. Supplementary Figures and Tables

1.1 Supplementary Figures

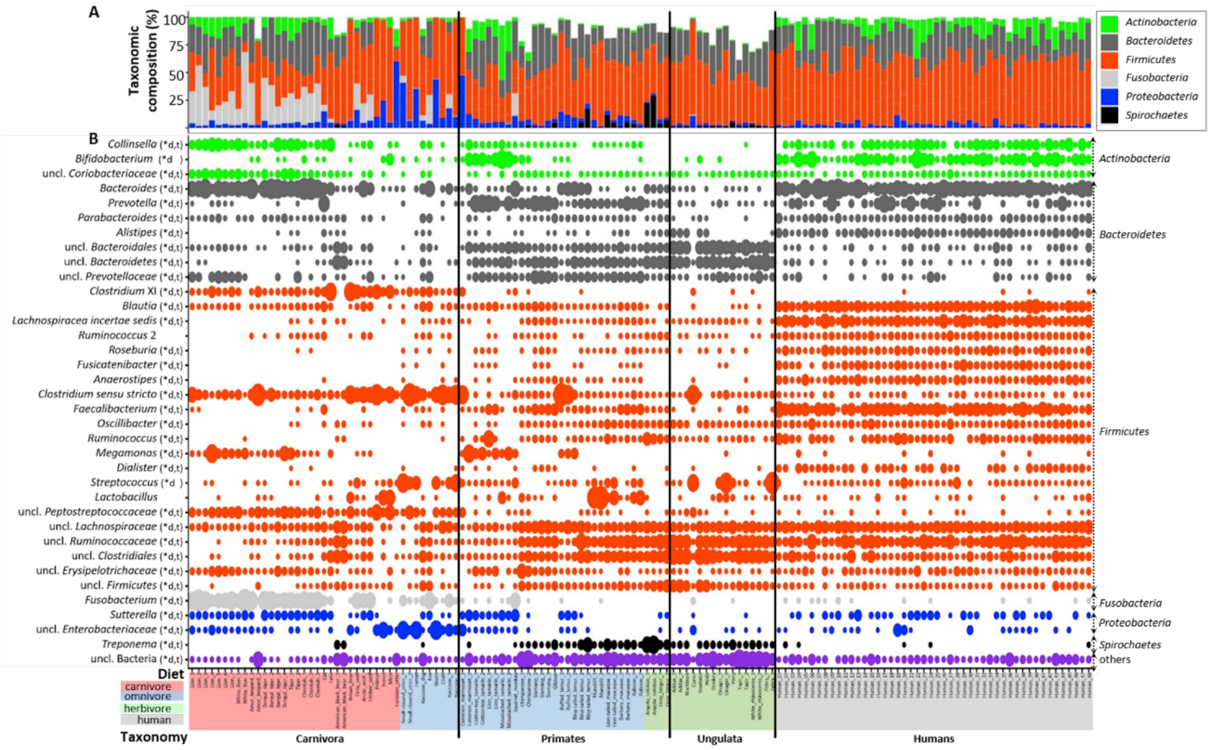

Supplementary Figure 1. Total bacterial community composition in individual mammals. Bar graph in panel A depicts the relative taxonomic composition of the total bacterial community whereas the bubble plot in panel B highlights the relative abundance of each OTU. Both graphs show the results for all animals in the dietary groups carnivores, omnivores, and herbivores (colored in red, blue, and green, respectively) as well as the taxonomic groups Carnivora, Primates and Ungulata (divided by black bars). On the right, human samples (colored in gray) are presented for comparison. * denotes significant difference in relative abundance between dietary (d) and taxonomic (t) groups as calculated by Kruskal-Wallis test. Only taxa with mean abundance of >1% are shown.

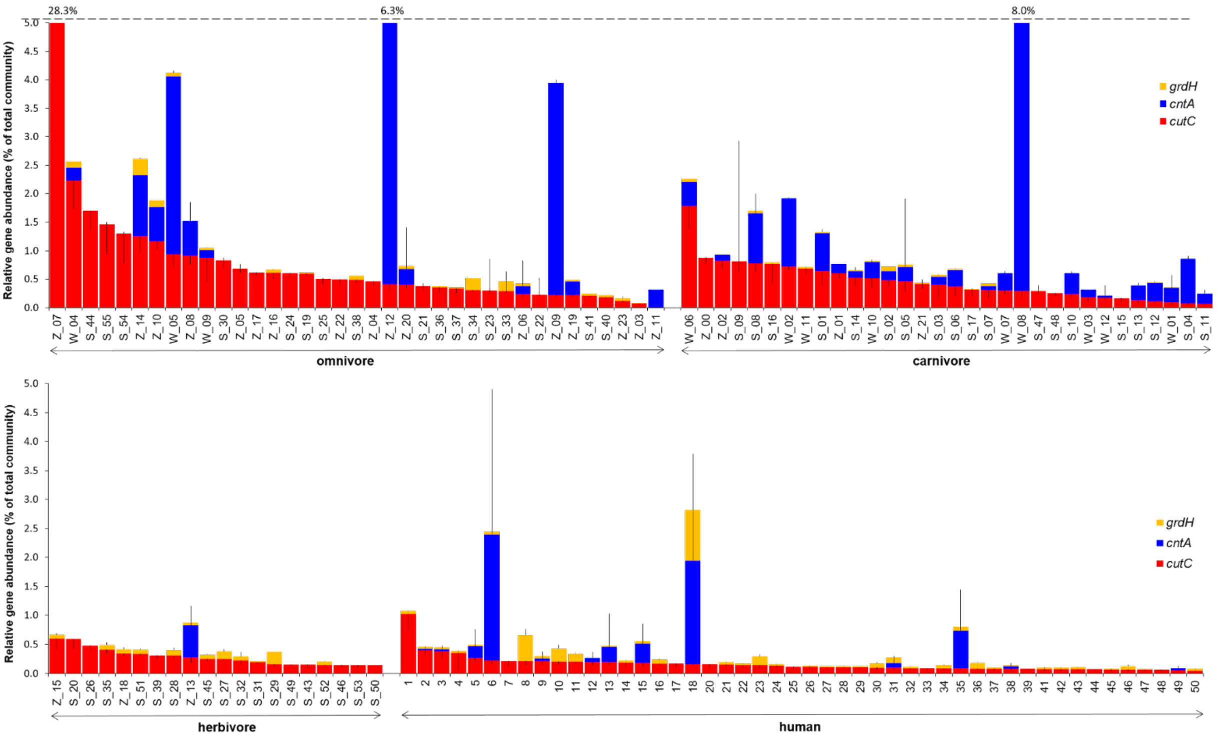

Supplementary Figure 2. Gene abundance of *cutC* (red), *cntA* (blue) and *grdH* (orange) in fecal samples from mammals. Gene abundance are shown relative to the total amount of 16S rRNA gene copies of a sample. Samples are sorted by descending quantity of *cutC*, error bars represent standard deviation on triplicate measurements.

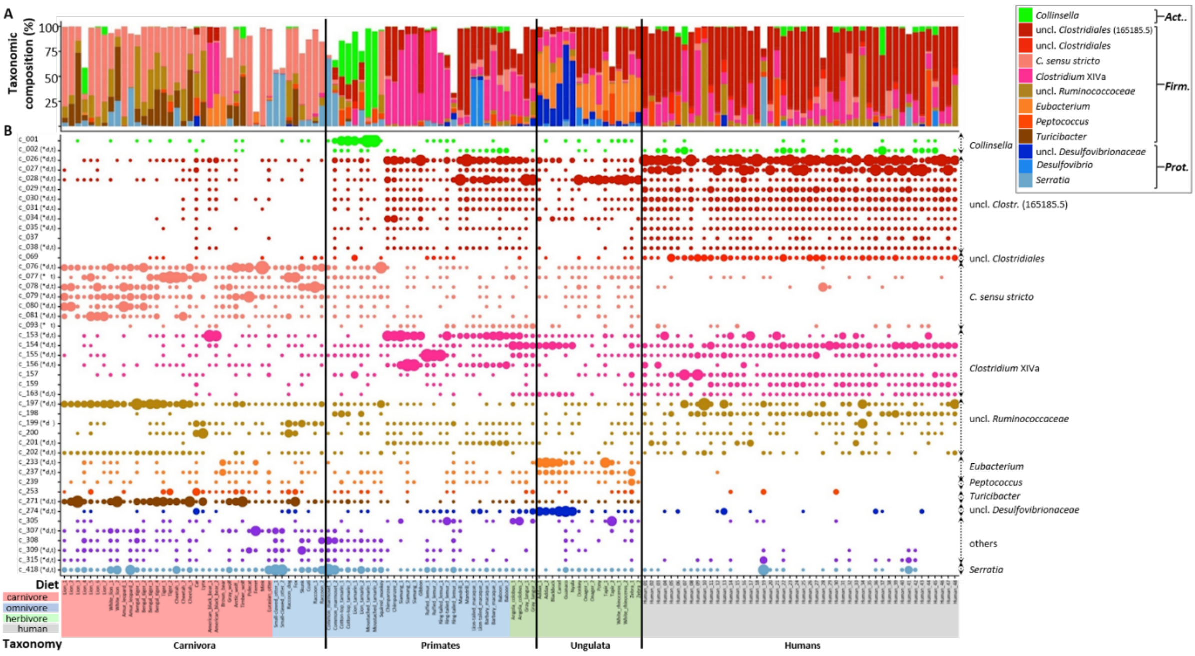

Supplementary Figure 3. *CutC* gene type abundance and diversity in individual mammals. Bar graph in panel **A** depicts the relative abundance of *cutC* gene types sorted by the assumed bacterial host whereas the bubble plot in panel **B** highlights the relative abundance of each gene type. Both graphs show the results for all animals in the dietary groups carnivores, omnivores, and herbivores (colored in red, blue, and green, respectively) as well as the taxonomic groups Carnivora, Primates and Ungulata (divided by black bars). On the right, human samples (colored in gray) are presented for comparison. * denotes significant difference in relative abundance between dietary (d) and taxonomic (t) groups as calculated by Kruskal-Wallis test. Only taxa with mean abundance of >1% are shown.

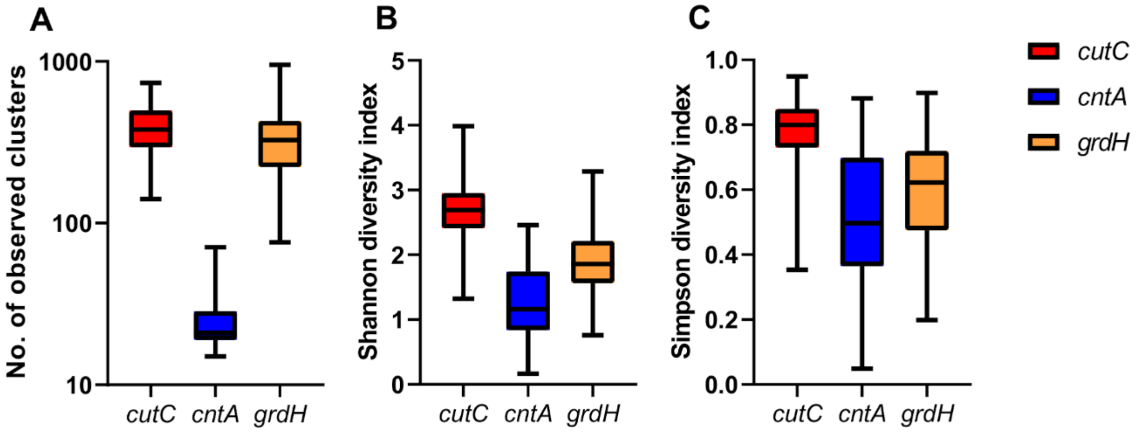

Supplementary Figure 4. Diversity indices of functional gene communities (*cutC*, *cntA* and *grdH*) in all samples analyzed. Panel (A) shows die number of observed clusters in those communities, (B) visualises the Shannon diversity index (H) and (C) depicts the inverse Simpson diversity index (1/λ).

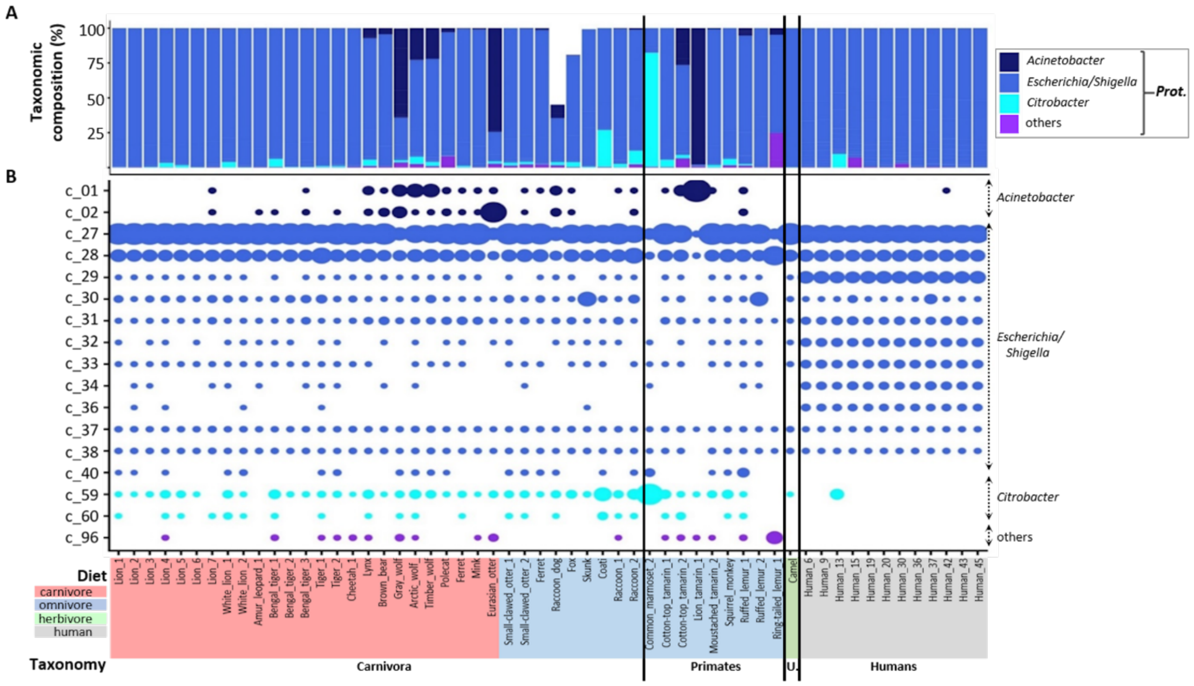

Supplementary Figure 5. ***CntA*** **gene type abundance and diversity in individual mammals.** Bar graph in panel **A** depicts the relative abundance of *cntA* gene types sorted by the assumed bacterial host whereas the bubble plot in panel **B** highlights the relative abundance of each gene type. Both graphs show the results for all animals in the dietary groups carnivores, omnivores, and herbivores (colored in red, blue, and green, respectively) as well as the taxonomic groups Carnivora, Primates and Ungulata (divided by black bars). On the right, human samples (colored in gray) are presented for comparison. * denotes significant difference in relative abundance between dietary (d) and taxonomic (t) groups as calculated by Kruskal-Wallis test. Only taxa with mean abundance of >1% are shown.

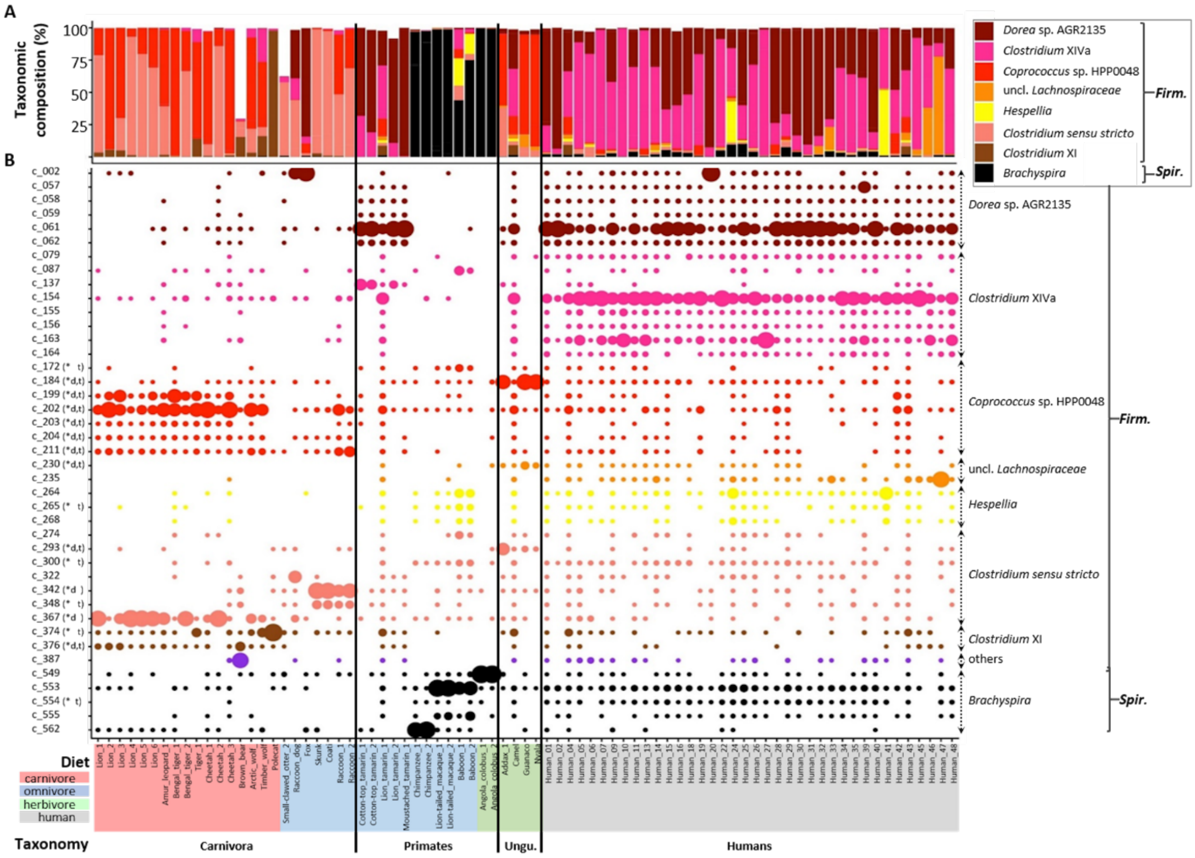

Supplementary Figure 6. *GrdH* gene type abundance and diversity in individual mammals. Bar graph in panel **A** depicts the relative abundance of *grdH* gene types sorted by the assumed bacterial host whereas the bubble plot in panel **B** highlights the relative abundance of each gene type. Both graphs show the results for all animals in the dietary groups carnivores, omnivores, and herbivores (colored in red, blue, and green, respectively) as well as the taxonomic groups Carnivora, Primates and Ungulata (divided by black bars). On the right, human samples (colored in gray) are presented for comparison. * denotes significant difference in relative abundance between dietary (d) and taxonomic (t) groups as calculated by Kruskal-Wallis test. Only taxa with mean abundance of >1% are shown.

1.2 Supplementary Tables

Supplementary Table 1. Effects of different types of diet on TMA(O) levels.

Diet	Study (reference)	Subject	Influence on TMA(O)
**Lean seafood**	(Schmedes et al., 2019)	human	TMA ↑ in feces
**Fish**	(Zeisel and DaCosta, 1986)	human	TMAO ↑ in urine
	(Svensson et al., 1994)	human	TMA(O) ↑ in urine from Swedish
	(Zuppi et al., 1998)	human	TMAO ↑ in urine from Norwegian
	(Zhang et al., 1999)	human	TMA(O) ↑ in urine
	(Lenz et al., 2004)	human	TMAO ↑ in urine from Swedish
	(Dumas et al., 2006)	human	TMAO ↑ in urine from Japanese
	(Dambrova et al., 2013)	human	TMAO ↑ in plasma
	(Cho et al., 2017)	human	TMAO ↑ in urine and plasma
**Eggs**	(Tang et al., 2013)	human	TMAO ↑ in plasma
	(West et al., 2014)	human	no change of TMAO in plasma after 8-wk of 6 eggs/wk
	(Miller et al., 2014)	human	TMAO ↑ in urine and plasma after ≥2 eggs
	(DiMarco et al., 2017)	human	no change of TMAO in plasma after 4 wk of 3 eggs/day
**Vegetarian**	(Xu et al., 2010)	human	TMAO ↓ in urine
	(Koeth et al., 2013)	human	TMAO ↓ in plasma
	(Zhu et al., 2017)	human	TMAO ↓ in plasma
	(Koeth et al., 2018)	human	TMAO ↓ in plasma
**High meat**	(Stella et al., 2006)	human	TMAO ↑ in urine
	(Wang et al., 2018)	human	TMA(O) ↑ in plasma

**Red meat**	(Van Hecke et al., 2016)<br>(Wang et al., 2018)	rat<br>human	TMAO ↑ in urine<br>TMAO ↑ in urine
**Resistant starch**	(Bergeron et al., 2016)<br>(Vital et al., 2018)<br>(Koay et al., 2019)	human<br>human<br>mouse	TMAO ↑ in plasma<br>No change of TMA synthesis pathways in metagenomes<br>TMAO ↑ in plasma
**High fat**	(Gao et al., 2014)<br>(Boutagy et al., 2015a)<br>(Boutagy et al., 2015b)<br>(Sun et al., 2017)<br>(Wang et al., 2018)<br>(Park et al., 2019)	mouse<br>human<br>human<br>mouse<br>human<br>human	No change of TMAO in serum<br>TMAO ↑ in plasma<br>TMAO ↑ in plasma (postprandial)<br>TMAO ↑ in plasma<br>No change of TMA(O) in plasma<br>TMAO ↑ in plasma
**Low fat**	(Park et al., 2019)	human	TMAO ↓ in plasma
**High protein**	(Rasmussen et al., 2012)	human	TMAO ↑ in urine
**Low protein**	(Mafra et al., 2018)	human	TMAO ↓ in plasma
**Western**	(Chen et al., 2017)	mouse	TMAO ↑ in plasma
**Pistachios**	(Hernández-Alonso et al., 2017)	human	TMAO ↓ in urine
**Histidine**	(Du et al., 2017)	human	TMAO ↑ in plasma/ urine

**Soy**	(Solanky et al., 2005)	human	TMAO	↑ in urine
**Glycemic-load carbs**	(Barton et al., 2015)	human	TMAO	↑ in plasma
**Allicin from garlic**	(Wu et al., 2015)	mouse	TMAO	↓ in plasma

References

Barton, S., Navarro, S. L., Buas, M. F., Schwarz, Y., Gu, H., Djukovic, D., et al. (2015). Targeted plasma metabolome response to variations in dietary glycemic load in a randomized, controlled, crossover feeding trial in healthy adults. Food Funct. 6, 2949–2956. doi:10.1039/c5fo00287g.

Bergeron, N., Williams, P. T., Lamendella, R., Faghihnia, N., Grube, A., Li, X., et al. (2016). Diets high in resistant starch increase plasma levels of trimethylamine-N-oxide, a gut microbiome metabolite associated with CVD risk. Br. J. Nutr. 116, 2020–2029. doi:10.1017/S0007114516004165.

Boutagy, N. E., Neilson, A. P., Osterberg, K. L., Smithson, A. T., Englund, T. R., Davy, B. M., et al. (2015a). Probiotic supplementation and trimethylamine-N-oxide production following a high-fat diet. Obesity 23, 2357–2363. doi:10.1002/oby.21212.

Boutagy, N. E., Neilson, A. P., Osterberg, K. L., Smithson, A. T., Englund, T. R., Davy, B. M., et al. (2015b). Short-term high-fat diet increases postprandial trimethylamine-N-oxide in humans. Nutr. Res. 35, 858–864. doi:10.1016/j.nutres.2015.07.002.

Chen, K., Zheng, X., Feng, M., Li, D., and Zhang, H. (2017). Gut microbiota-dependent metabolite trimethylamine N-oxide contributes to cardiac dysfunction in western diet-induced obese mice. Front. Physiol. 8, 139. doi:10.3389/FPHYS.2017.00139.

Cho, C. E., Taesuwan, S., Malysheva, O. V., Bender, E., Tulchinsky, N. F., Yan, J., et al. (2017). Trimethylamine-N-oxide (TMAO) response to animal source foods varies among healthy young men and is influenced by their gut microbiota composition: A randomized controlled trial. Mol. Nutr. Food Res. 1600324, 1–12. doi:10.1002/mnfr.201600324.

Dambrova, M., Skapare-makarova, E., Konrade, I., Pugovics, O., Grinberga, S., Tirzite, D., et al. (2013). Meldonium decreases the diet-increased plasma levels of trimethylamine n-oxide, a metabolite associated with atherosclerosis. J. Clin. Pharmacol. 53, 1095–1098. doi:10.1002/jcph.135.

DiMarco, D. M., Missimer, A., Murillo, A. G., Lemos, B. S., Malysheva, O. V., Caudill, M. A., et al. (2017). Intake of up to 3 eggs/day increases HDL cholesterol and plasma choline while plasma trimethylamine-N-oxide is unchanged in a healthy population. Lipids. doi:10.1007/s11745-017-4230-9.

Du, S., Sun, S., Liu, L., Zhang, Q., Guo, F., Li, C., et al. (2017). Effects of histidine supplementation on global serum and urine 1H NMR-based metabolomics and serum amino acid profiles in obese women from a randomized controlled study. J. Proteome Res. 16, 2221–2230. doi:10.1021/acs.jproteome.7b00030.

Dumas, M.-E., Maibaum, E. C., Teague, C., Ueshima, H., Zhou, B., Lindon, J. C., et al. (2006). Assessment of analytical reproducibility of1H NMR spectroscopy based metabonomics for large-scale epidemiological research: The INTERMAP study. Anal. Chem. 78, 2199–2208. doi:10.1021/ac0517085.

Gao, X., Liu, X., Xu, J., Xue, C., Xue, Y., and Wang, Y. (2014). Dietary trimethylamine N-oxide exacerbates impaired glucose tolerance in mice fed a high fat diet. J. Biosci. Bioeng. 118, 476–481. doi:10.1016/j.jbiosc.2014.03.001.

Hernández-Alonso, P., Cañueto, D., Giardina, S., Salas-Salvadó, J., Cañellas, N., Correig, X., et al. (2017). Effect of pistachio consumption on the modulation of urinary gut microbiota-related metabolites in prediabetic subjects. J. Nutr. Biochem. 45, 48–53.

Koay, Y. C., Wali, J. A., Luk, A. W. S., Macia, L., Cogger, V. C., Pulpitel, T. J., et al. (2019). Ingestion of resistant starch by mice markedly increases microbiome-derived metabolites. FASEB J. doi:10.1096/fj.201900177R.

Koeth, R. A., Lam-Galvez, B. R., Kirsop, J., Wang, Z., Levison, B. S., Gu, X., et al. (2018). l-Carnitine in omnivorous diets induces an atherogenic gut microbial pathway in humans. J. Clin. Invest. 129, 373–387. doi:10.1172/JCI94601.

Koeth, R. A., Wang, Z., Levison, B. S., Buffa, J. A., Org, E., Sheehy, B. T., et al. (2013). Intestinal microbiota metabolism of L-carnitine, a nutrient in red meat, promotes atherosclerosis. Nat. Med. 19, 576–85. doi:10.1038/nm.3145.

Lenz, E. M., Bright, J., Wilson, I. D., Hughes, A., Morrisson, J., Lindberg, H., et al. (2004). Metabonomics, dietary influences and cultural differences: A 1H NMR-based study of urine samples obtained from healthy British and Swedish subjects. J. Pharm. Biomed. Anal. 36, 841–849. doi:10.1016/j.jpba.2004.08.002.

Mafra, D., Borges, N. A., Ferreira Madeiros de Franca Cardozo, L., Anjos, J. S., Black, P., Moraes, C., et al. (2018). Red meat intake in chronic kidney disease patients: Two sides of the coin. Nutrition 46, 26–32. doi:10.1016/j.nut.2017.08.015.

Miller, C. A., Corbin, K. D., Costa, K., Zhang, S., Zhao, X., Galanko, J. A., et al. (2014). Effect of egg ingestion on trimethylamine-N-oxide production in humans: a randomized, controlled, dose-response study. Am. J. Clin. Nutr. 100, 778–786. doi:10.3945/ajcn.114.087692.1.

Park, J. E., Miller, M., Rhyne, J., Wang, Z., and Hazen, S. L. (2019). Differential effect of short-term popular diets on TMAO and other cardio-metabolic risk markers. Nutr. Metab. Cardiovasc. Dis. 29, 513–517. doi:10.1016/j.numecd.2019.02.003.

Rasmussen, L. G., Winning, H., Savorani, F., Toft, H., Larsen, T. M., Dragsted, L. O., et al. (2012). Assessment of the effect of high or low protein diet on the human urine metabolome as measured by NMR. Nutrients 4, 112–131. doi:10.3390/nu4020112.

Schmedes, M., Brejnrod, A. D., Aadland, E. K., Kiilerich, P., Kristiansen, K., Jacques, H., et al. (2019). The effect of lean-seafood and non-seafood diets on fecal metabolites and gut microbiome: results from a randomized crossover intervention study. Mol. Nutr. Food Res. 63, 1–8. doi:10.1002/mnfr.201700976.

Solanky, K. S., Bailey, N. J., Beckwith-Hall, B. M., Bingham, S., Davis, A., Holmes, E., et al. (2005). Biofluid 1H NMR-based metabonomic techniques in nutrition research - Metabolic effects of dietary isoflavones in humans. J. Nutr. Biochem. 16, 236–244. doi:10.1016/j.jnutbio.2004.12.005.

Stella, C., Beckwith-Hall, B., Cloarec, O., Holmes, E., Lindon, J. C., Powell, J., et al. (2006). Susceptibility of human metabolic phenotypes to dietary modulation. J. Proteome Res. 5, 2780–2788. doi:10.1021/pr060265y.

Sun, G., Yin, Z., Liu, N., Bian, X., Yu, R., Su, X., et al. (2017). Gut microbial metabolite TMAO contributes to renal dysfunction in a mouse model of diet-induced obesity. Biochem. Biophys. Res. Commun. 493, 964–970. doi:10.1016/j.bbrc.2017.09.108.

Svensson, B.-G., Åkesson, B., Nilsson, A., and Paulsson, K. (1994). Urinary excretion of methylamines in men with varying intake of fish from the baltic sea. J. Toxicol. Environ. Health 41, 411–420. doi:10.1080/15287399409531853.

Tang, W. H. W., Wang, Z., Levison, B. S., Koeth, R. a, Britt, E. B., Fu, X., et al. (2013). Intestinal microbial metabolism of phosphatidylcholine and cardiovascular risk. N. Engl. J. Med. 368, 1575–1584. doi:10.1056/NEJMoa1109400.

Van Hecke, T., Jakobsen, L. M. A., Vossen, E., Guéraud, F., De Vos, F., Pierre, F., et al. (2016). Short-term beef consumption promotes systemic oxidative stress, TMAO formation and inflammation in rats, and dietary fat content modulates these effects. Food Funct. 7, 3760–3771. doi:10.1039/c6fo00462h.

Vital, M., Howe, A., Bergeron, N., Krauss, R. M., Jansson, J. K., and Tiedje, J. M. (2018). Metagenomic insights into the degradation of resistant starch by human gut microbiota. Appl. Environ. Microbiol. 84, 1–13. doi:10.1128/aem.01562-18.

Wang, Z., Bergeron, N., Levison, B. S., Li, X. S., Chiu, S., Jia, X., et al. (2018). Impact of chronic dietary red meat, white meat, or non-meat protein on trimethylamine N-oxide metabolism and renal excretion in healthy men and women. Eur. Heart J. 00, 1–13. doi:10.1093/eurheartj/ehy799.

West, A. A., Shih, Y., Wang, W., Oda, K., Jaceldo-Siegl, K., Sabaté, J., et al. (2014). Egg n-3 fatty acid composition modulates biomarkers of choline metabolism in free-living lacto-ovo-vegetarian women of reproductive age. J. Acad. Nutr. Diet. 114, 1594–1600. doi:10.1016/j.jand.2014.02.012.

Wu, W. K., Panyod, S., Ho, C. T., Kuo, C. H., Wu, M. S., and Sheen, L. Y. (2015). Dietary allicin reduces transformation of L-carnitine to TMAO through impact on gut microbiota. J. Funct. Foods 15, 408–417. doi:10.1016/j.jff.2015.04.001.

Xu, J., Yang, S., Cai, S., Dong, J., Li, X., and Chen, Z. (2010). Identification of biochemical changes in lactovegetarian urine using 1 H NMR spectroscopy and pattern recognition. Anal. Bioanal. Chem. 396, 1451–1463. doi:10.1007/s00216-009-3338-z.

Zeisel, S. H., and DaCosta, K. A. (1986). Increase in human exposure to methylamine precursors of N-nitrosamines after eating fish. Cancer Res. 46, 6136–6138.

Zhang, A. Q., Mitchell, S. C., and Smith, R. L. (1999). Dietary precursors of trimethylamine in man: A pilot study. Food Chem. Toxicol. 37, 515–520. doi:10.1016/S0278-6915(99)00028-9.

Zhu, W., Wang, Z., Tang, W. H. W., and Hazen, S. L. (2017). Gut microbe-generated trimethylamine N-oxide from dietary choline is prothrombotic in subjects. Circulation 135, 1671–1673. doi:10.1161/CIRCULATIONAHA.116.025338.

Zuppi, C., Messana, I., Forni, F., Ferrari, F., Rossi, C., and Giardina, B. (1998). Influence of feeding on metabolite excretion evidenced by urine 1H NMR spectral profiles: A comparison between subjects living in Rome and subjects living at arctic latitudes (Svaldbard). Clin. Chim. Acta 278, 75–79. doi:10.1016/S0009-8981(98)00132-6.

Supplementary Table 2. Diet and taxonomy of the samples included in the study. Abbreviations: C, carnivores; H, herbivores; O, omnivores.

Sample ID	Class	Order	Family	(Sub)species (Common name)	Location	Diet
**S_01**	Mammalia	Carnivora	Felidae	Lion	Seregenti Park, Hodenhagen	C
**S_02**	Mammalia	Carnivora	Felidae	Lion	Seregenti Park, Hodenhagen	C
**S_03**	Mammalia	Carnivora	Felidae	Lion	Seregenti Park, Hodenhagen	C
**S_04**	Mammalia	Carnivora	Felidae	Lion	Seregenti Park, Hodenhagen	C
**S_05**	Mammalia	Carnivora	Felidae	Lion	Seregenti Park, Hodenhagen	C
**S_06**	Mammalia	Carnivora	Felidae	Lion	Seregenti Park, Hodenhagen	C
**S_07**	Mammalia	Carnivora	Felidae	Lion	Seregenti Park, Hodenhagen	C
**S_10**	Mammalia	Carnivora	Felidae	White lion	Seregenti Park, Hodenhagen	C
**S_11**	Mammalia	Carnivora	Felidae	White lion	Seregenti Park, Hodenhagen	C
**S_08**	Mammalia	Carnivora	Felidae	Amur leopard	Seregenti Park, Hodenhagen	C
**S_09**	Mammalia	Carnivora	Felidae	Amur leopard	Seregenti Park, Hodenhagen	C
**S_12**	Mammalia	Carnivora	Felidae	Bengal tiger	Seregenti Park, Hodenhagen	C
**S_13**	Mammalia	Carnivora	Felidae	Bengal tiger	Seregenti Park, Hodenhagen	C
**S_14**	Mammalia	Carnivora	Felidae	Bengal tiger	Seregenti Park, Hodenhagen	C
**S_15**	Mammalia	Carnivora	Felidae	Bengal tiger	Seregenti Park, Hodenhagen	C
**Z_01**	Mammalia	Carnivora	Felidae	Tiger	Arche Noah Zoo, Braunschweig	C
**Z_02**	Mammalia	Carnivora	Felidae	Tiger	Arche Noah Zoo, Braunschweig	C
**S_16**	Mammalia	Carnivora	Felidae	Cheetah	Seregenti Park, Hodenhagen	C
**S_17**	Mammalia	Carnivora	Felidae	Cheetah	Seregenti Park, Hodenhagen	C
**Z_21**	Mammalia	Carnivora	Felidae	Cheetah	Arche Noah Zoo, Braunschweig	C
**Z_00**	Mammalia	Carnivora	Felidae	Cat	House cat, Braunschweig	C
**W_12**	Mammalia	Carnivora	Felidae	Lynx	Wisentgehege Springe, Hannover	C
**S_47**	Mammalia	Carnivora	Ursidae	American black bear	Seregenti Park, Hodenhagen	C
**S_48**	Mammalia	Carnivora	Ursidae	American black bear	Seregenti Park, Hodenhagen	C
**W_06**	Mammalia	Carnivora	Ursidae	Brown bear	Wisentgehege Springe, Hannover	C
**W_07**	Mammalia	Carnivora	Canidae	Gray wolf	Wisentgehege Springe, Hannover	C

**W_10**	Mammalia	Carnivora	Canidae	Arctic wolf	Wisentgehege Springe, Hannover	C
**W_11**	Mammalia	Carnivora	Canidae	Timber wolf	Wisentgehege Springe, Hannover	C
**W_01**	Mammalia	Carnivora	Mustelidae	Polecat	Wisentgehege Springe, Hannover	C
**W_02**	Mammalia	Carnivora	Mustelidae	Ferret	Wisentgehege Springe, Hannover	C
**W_03**	Mammalia	Carnivora	Mustelidae	Mink	Wisentgehege Springe, Hannover	C
**W_08**	Mammalia	Carnivora	Mustelidae	Eurasian otter	Wisentgehege Springe, Hannover	C
**Z_09**	Mammalia	Carnivora	Mustelidae	Small-clawed otter	Arche Noah Zoo, Braunschweig	O
**Z_19**	Mammalia	Carnivora	Mustelidae	Small-clawed otter	Arche Noah Zoo, Braunschweig	O
**Z_11**	Mammalia	Carnivora	Mustelidae	Ferret	Arche Noah Zoo, Braunschweig	O
**W_04**	Mammalia	Carnivora	Canidae	Raccoon dog	Wisentgehege Springe, Hannover	O
**W_09**	Mammalia	Carnivora	Canidae	Fox	Wisentgehege Springe, Hannover	O
**Z_12**	Mammalia	Carnivora	Mephitidae	Skunk	Arche Noah Zoo, Braunschweig	O
**Z_10**	Mammalia	Carnivora	Procyonidae	Coati	Arche Noah Zoo, Braunschweig	O
**Z_14**	Mammalia	Carnivora	Procyonidae	Raccoon	Arche Noah Zoo, Braunschweig	O
**W_05**	Mammalia	Carnivora	Procyonidae	Raccoon	Wisentgehege Springe, Hannover	O
**Z_07**	Mammalia	Primates	Callitrichidae	Common marmoset	Arche Noah Zoo, Braunschweig	O
**Z_17**	Mammalia	Primates	Callitrichidae	Common marmoset	Arche Noah Zoo, Braunschweig	O
**Z_06**	Mammalia	Primates	Callitrichidae	Cotton-top tamarin	Arche Noah Zoo, Braunschweig	O
**Z_23**	Mammalia	Primates	Callitrichidae	Cotton-top tamarin	Arche Noah Zoo, Braunschweig	O
**Z_03**	Mammalia	Primates	Callitrichidae	Lion tamarin	Arche Noah Zoo, Braunschweig	O
**Z_20**	Mammalia	Primates	Callitrichidae	Lion tamarin	Arche Noah Zoo, Braunschweig	O
**Z_04**	Mammalia	Primates	Callitrichidae	Moustached tamarin	Arche Noah Zoo, Braunschweig	O
**Z_16**	Mammalia	Primates	Callitrichidae	Moustached tamarin	Arche Noah Zoo, Braunschweig	O
**Z_08**	Mammalia	Primates	Cebidae	Squirrel monkey	Arche Noah Zoo, Braunschweig	O
**S_19**	Mammalia	Primates	Hominidae	Chimpanzee	Seregenti Park, Hodenhagen	O
**S_38**	Mammalia	Primates	Hominidae	Chimpanzee	Seregenti Park, Hodenhagen	O
**S_23**	Mammalia	Primates	Hylobatidae	Siamang	Seregenti Park, Hodenhagen	O
**S_24**	Mammalia	Primates	Hylobatidae	Siamang	Seregenti Park, Hodenhagen	O
**S_25**	Mammalia	Primates	Hylobatidae	Siamang	Seregenti Park, Hodenhagen	O
**S_30**	Mammalia	Primates	Hylobatidae	Gibbon	Seregenti Park, Hodenhagen	O

**S_54**	Mammalia	Primates	Lemuridae	Ruffed lemur	Seregenti Park, Hodenhagen	O
**S_55**	Mammalia	Primates	Lemuridae	Ruffed lemur	Seregenti Park, Hodenhagen	O
**S_44**	Mammalia	Primates	Lemuridae	Ring-tailed lemur	Seregenti Park, Hodenhagen	O
**Z_05**	Mammalia	Primates	Lemuridae	Ring-tailed lemur	Arche Noah Zoo, Braunschweig	O
**Z_22**	Mammalia	Primates	Lemuridae	Ring-tailed lemur	Arche Noah Zoo, Braunschweig	O
**S_21**	Mammalia	Primates	Cercopithecidae	Mandrill	Seregenti Park, Hodenhagen	O
**S_22**	Mammalia	Primates	Cercopithecidae	Mandrill	Seregenti Park, Hodenhagen	O
**S_33**	Mammalia	Primates	Cercopithecidae	Lion-tailed macaque	Seregenti Park, Hodenhagen	O
**S_34**	Mammalia	Primates	Cercopithecidae	Lion-tailed macaque	Seregenti Park, Hodenhagen	O
**S_36**	Mammalia	Primates	Cercopithecidae	Barbary macaque	Seregenti Park, Hodenhagen	O
**S_37**	Mammalia	Primates	Cercopithecidae	Barbary macaque	Seregenti Park, Hodenhagen	O
**S_40**	Mammalia	Primates	Cercopithecidae	Baboon	Seregenti Park, Hodenhagen	O
**S_41**	Mammalia	Primates	Cercopithecidae	Baboon	Seregenti Park, Hodenhagen	O
**S_27**	Mammalia	Primates	Cercopithecidae	Angola colobus	Seregenti Park, Hodenhagen	H
**S_29**	Mammalia	Primates	Cercopithecidae	Angola colobus	Seregenti Park, Hodenhagen	H
**S_20**	Mammalia	Primates	Cercopithecidae	Gray langur	Seregenti Park, Hodenhagen	H
**S_32**	Mammalia	Primates	Cercopithecidae	Gray langur	Seregenti Park, Hodenhagen	H
**S_28**	Mammalia	Artiodactyla	Bovidae	Addax	Seregenti Park, Hodenhagen	H
**S_51**	Mammalia	Artiodactyla	Bovidae	Addax	Seregenti Park, Hodenhagen	H
**S_31**	Mammalia	Artiodactyla	Bovidae	Blackbuck	Seregenti Park, Hodenhagen	H
**Z_13**	Mammalia	Artiodactyla	Camelidae	Camel	Arche Noah Zoo, Braunschweig	H
**S_45**	Mammalia	Artiodactyla	Camelidae	Guanaco	Seregenti Park, Hodenhagen	H
**S_35**	Mammalia	Artiodactyla	Bovidae	Nyala	Seregenti Park, Hodenhagen	H
**Z_18**	Mammalia	Perissodactyla	Equidae	Donkey	Arche Noah Zoo, Braunschweig	H
**S_26**	Mammalia	Perissodactyla	Equidae	Onager	Seregenti Park, Hodenhagen	H
**S_39**	Mammalia	Perissodactyla	Equidae	Onager	Seregenti Park, Hodenhagen	H
**Z_15**	Mammalia	Perissodactyla	Equidae	Pony	Arche Noah Zoo, Braunschweig	H
**S_43**	Mammalia	Perissodactyla	Tapiridae	Tapir	Seregenti Park, Hodenhagen	H
**S_46**	Mammalia	Perissodactyla	Tapiridae	Tapir	Seregenti Park, Hodenhagen	H
**S_52**	Mammalia	Perissodactyla	Rhinocerotidae	White rhinoceros	Seregenti Park, Hodenhagen	H

**S_53**	Mammalia	Perissodactyla	Rhinocerotidae	White rhinoceros	Seregenti Park, Hodenhagen	H
**S_49**	Mammalia	Perissodactyla	Equidae	Zebra	Seregenti Park, Hodenhagen	H
**S_50**	Mammalia	Perissodactyla	Equidae	Zebra	Seregenti Park, Hodenhagen	H

Supplementary Table 3. Sequences of primers targeting *cutC*, *cntA* and *grdH* are shown. Bold letters highlight the degenerate bases (cutC_F yields 32 combinations, cutC_R 16; cntA_F 32; cntA_R 512; grdH_F 32; grdH_R 16). The expected size of amplified product excluding primers is 275 bp (*cutC*), 249 bp (*cntA*) and 227 bp (*grdH*). * from Rath *et al.* (2017)

	**Forward primer (5′➔ 3′)**	**Reverse primer (5′➔ 3′)**
cutC*	TT**Y**GC**I**GG**I**TA**Y**CA**R**CC**N**TT	TG**N**GG**Y**TC**I**AC**R**CA**I**CCCAT
cntA*	TA**Y**CA**Y**GC**I**TGG**R**C**I**TT**Y**AA**R**CT	**R**CAGTG**R**TA**R**CA**Y**TC**S**A**KR**TAGTT**R**TC**R**AC
grdH	AT**W**CA**R**TC**I**GC**W**TC**I**GC**N**AC	GT**I**GT**W**CC**N**GT**W**CC**I**AC**I**GT

Supplementary Table 4. Relative importance values (Imp.) of specific features based on Random Forests analyses for diet and taxonomy (Tax.) model.

16S rDNA				*cutC*				*grdH*			
**Diet**	**Imp.**	**Tax.**	**Imp.**	**Diet**	**Imp.**	**Tax.**	**Imp.**	**Diet**	**Imp.**	**Tax.**	**Imp.**
Bacteroides	100.00	*Roseburia*	100.00	159	100.00	069	100.00	554	100.00	061	100.00
Dialister	99.58	*Clostridium* XI	89.89	069	90.61	028	88.15	061	87.87	163	87.98
Roseburia	97.34	*Bifidobacterium*	83.11	079	90.13	159	80.25	367	57.53	057	71.44
Alistipes	91.04	*Treponema*	82.50	028	89.39	198	68.02	164	56.65	554	69.22
Ruminococcus 2	76.98	*Ruminococcus* 2	67.92	272	76.44	272	63.76	163	49.04	211	50.41
Prevotella	68.08	*Alistipes*	67.69	037	51.77	076	57.30	553	43.96	164	39.12
Oscillibacter	64.01	*Prevotella*	65.42	198	51.25	079	55.15	376	42.89	235	15.76
Clostridium XI	53.93	*Bacteroides*	65.40	076	49.13	037	45.34	230	33.14	230	13.72
Treponoma	53.55	*Ruminococcus*	63.03	202	37.02	034	29.72	199	31.48	264	11.05
uncl. *Firmicutes*	51.48	*Fusobacterium*	55.06	233	35.62	233	27.76	057	28.05	555	10.69
uncl. *Prevotellaceae*	47.17	*Oscillibacter*	54.81	274	33.69	002	22.19	211	2.62	367	5.99
Bifidobacterium	36.80	*C. sensu stricto*	41.75	201	29.82	274	20.15	235	2.09	203	4.97
Ruminococcus	32.30	*Collinsella*	34.65	081	28.84	092	17.10	300	0.00	293	0.00
uncl. *Coriobacteriaceae*	32.27	uncl. *Prevotellaceae*	34.38	080	26.60	078	16.53				
Parabacteroides	32.04	*Dialister*	29.53	002	23.94	156	14.78				
Clostridium sensu stricto	31.02	*Parabacteroides*	28.59	308	23.77	153	14.52				
uncl. *Lachnospiraceae*	19.93	uncl. *Firmicutes*	14.00	156	22.94	202	14.29				
Sutterella	13.83	uncl. *Lachnospiraceae*	3.59	239	20.97	201	13.90				
uncl. *Erysipelotrichaceae*	7.03	*Sutterella*	2.46	034	17.22	157	13.81				
Megamonas	1.31	*Megamonas*	0.00	157	14.48	081	13.60				
